# Developing a Three-Dimensional (3D) Assessment Method for Clubfoot—A Study Protocol

**DOI:** 10.3389/fphys.2017.01098

**Published:** 2018-01-04

**Authors:** Balasankar Ganesan, Ameersing Luximon, Adel A. Al-Jumaily, Joanne Yip, Paul J. Gibbons, Alison Chivers

**Affiliations:** ^1^Institute of Textiles and Clothing, The Hong Kong Polytechnic University, Kowloon, Hong Kong; ^2^Department of FEIT, University of Technology Sydney, Ultimo, NSW, Australia; ^3^University of Sydney and Department of Orthopaedic Surgery, The Children's Hospital at Westmead, Sydney, NSW, Australia; ^4^Department of Physiotherapy, The Children's Hospital at Westmead, Sydney, NSW, Australia

**Keywords:** clubfoot assessment, 3D scanning, infrared thermography, automatic assessment method

## Abstract

**Background:** Congenital talipes equinovarus (CTEV) or clubfoot is a common pediatric congenital foot deformity that occurs 1 in 1,000 live births. Clubfoot is characterized by four types of foot deformities: hindfoot equinus; midfoot cavus; forefoot adductus; and hindfoot varus. A structured assessment method for clubfoot is essential for quantifying the initial severity of clubfoot deformity and recording the progress of clubfoot intervention.

**Aim:** This study aims to develop a three-dimensional (3D) assessment method to evaluate the initial severity of the clubfoot and monitor the structural changes of the clubfoot after each casting intervention. In addition, this study explores the relationship between the thermophysiological changes in the clubfoot at each stage of the casting intervention and in the normal foot.

**Methods:** In this study, a total of 10 clubfoot children who are <2 years old will be recruited. Also, the data of the unaffected feet of a total of 10 children with unilateral clubfoot will be obtained as a reference for normal feet. A Kinect 3D scanner will be used to collect the 3D images of the clubfoot and normal foot, and an Infrared thermography camera (IRT camera) will be used to collect the thermal images of the clubfoot. Three-dimensional scanning and IR imaging will be performed on the foot once a week before casting. In total, 6–8 scanning sessions will be performed for each child participant. The following parameters will be calculated as outcome measures to predict, monitor, and quantify the severity of the clubfoot: Angles cross section parameters, such as length, width, and the radial distance; distance between selected anatomical landmarks, and skin temperature of the clubfoot and normal foot. The skin temperature will be collected on selected areas (forefoot, mid foot, and hindfoot) to find out the relationship between the thermophysiological changes in the clubfoot at each stage of the casting treatment and in the normal foot.

**Ethics:** The study has been reviewed and approved on 17 August 2016 by the Sydney Children's Hospitals Network Human Research Ethics Committee (SCHN HREC), Sydney, Australia. The Human Research Ethics Committee (HREC) registration number for this study is: HREC/16/SCHN/163.

## Introduction

Congenital talipes equinovarus (CTEV) or clubfoot is a common pediatric congenital foot deformity that occurs about 1 in 1,000 live births (Wynne-Davies, 1964; Shiels et al., 2007; Hussain et al., 2014; Meena et al., 2014; Smith et al., 2014), and this deformity is characterized by four components such as hindfoot equinus, midfoot cavus, forefoot adductus, and hindfoot varus (Solanki et al., 2010; Meena et al., 2014; Shabtai et al., 2014). In recent years, the “Ponseti method” (conservative treatment method) has been widely accepted worldwide to correct the clubfoot deformity. This method involves with manipulation, serial casting, percutaneous tendoachilles tenotomy, and bracing (Lykissas et al., 2013; Shabtai et al., 2014).

The assessment of clubfoot is very essential to evaluate the success rate of clubfoot treatment, to quantify the initial severity of the clubfoot, to monitor the progress of the clubfoot treatment, and to assess the relapses and treatment follow-up (Wainwright et al., 2002; Herd et al., 2004; Ramanathan et al., 2009; Ramanathan and Abboud, 2010; Bergerault et al., 2013; Cosma and Vasilescu, 2015; Lampasi et al., 2017). Therefore, many classification methods or clinical grading systems have been proposed to evaluate the clubfoot severities. For example, Harrold and Walker classification system (Harrold and Walker, 1983), Catterrall classification system (Catterall, 1991), Dimeglio classification system (Diméglio et al., 1995), Pirani scoring system (Pirani et al., 1999). In addition, the following imaging methods have been used as an objective assessment to quantify the severities of the clubfoot such as magnetic resonance imaging (MRI) (Cahuzac et al., 1999; Itohara et al., 2005; Duce et al., 2013), computed tomography (CT) (Reikeras et al., 2001; Ippolito et al., 2004; Farsetti et al., 2009), and radiography or X-ray methods (Ippolito et al., 2004; Baghdadi et al., 2017). However, several studies reported that there is no universal standard assessment method available at present to quantify the initial severity of the clubfoot or monitor the clubfoot treatment (Jain et al., 2001; Gigante et al., 2004; Dyer and Davis, 2006; Ramanathan et al., 2009; Ramanathan and Abboud, 2010; Yapp et al., 2012; Bhaskar and Patni, 2013). Because it is difficult to obtain the objective measurements from all these classification systems (Catterrall classification system, Pirani scoring, Dimeglio classification system, and Harrold and Walker classification system) due to subjective in nature (Wainwright et al., 2002; Jain et al., 2012, 2017; Fan et al., 2017). In view of imaging assessment methods, it provides objective details of severities of the clubfoot. But it has its own advantages and disadvantages for assessing the clubfoot. For instance, previous studies reported that the repeatability and accuracy of radiological measurements are questionable, and there is a difficulty to obtain the reliable information due to non-ossified tarsal bones in the new born babies (Simons, 1978; Hutchins et al., 1985; Uglow and Clarke, 2000; Gigante et al., 2004; Ramanathan and Abboud, 2010). Furthermore, positioning the newborn baby's feet accurately is difficult in the radiological examinations (Macnicol et al., 2000). The CT procedure emits radioactive waves, which may have adverse effects on humans (Zheng et al., 2001). On the other hand, MRI imaging methods can be used to visualize the soft tissue structure, bone ossification, and cartilage of the clubfoot (Richards and Dempsey, 2007), to assess the misalignment of the talonavicular joint (Kamegaya et al., 2001), and to assess the calcaneus adduction angle, navicular angle, talus neck angle, calcaneus shift index (Kamegaya et al., 2000). A study by Gutekunst (2012) suggested that using three dimensional (3D) modalities, such as MRI, would be useful for determining the severity of foot deformities by calculating bone to bone orientation analysis in the following conditions: club foot, rheumatoid arthritis, Charcot-Marie-Tooth disease, diabetic neuropathy, and Charcot neuroarthropathy. However, both MRI and CT are expensive procedures for quantifying and evaluating the severity of clubfoot at each stage of clubfoot casting intervention, and would require sedation or anesthesia for infants to make them calm or relax during the MRI procedures. Few studies used commercially available 3D scanner to evaluate the structure and shapes of the normal foot, and to evaluate the anthropometry of normal feet, including foot length and width, heel width, lateral malleolus height, foot width circumference, and lateral malleolus height (Lee et al., 2012; Ma and Luximon, 2014; Taha et al., 2014). To the best of our knowledge, only one study used the commercially available 3D scanner to quantify the structures of foot in rheumatoid arthritis patients (De Mits et al., 2012).

The most frequently used clinical scoring systems in the clinical settings are Pirani scoring and Dimeglio classification system, which are widely used to assess the severities of the clubfoot based on the physical aspects of the clubfoot (Hefti, 2007; Chu et al., 2010; Bergerault et al., 2013). Both clinical grading systems had been reported with excellent reliability and reproducibility (Flynn et al., 1999; Pirani et al., 2008; Shaheen et al., 2012). Also, the Pirani scoring system is gained more popularity than other scoring system for assessing the outcome of Ponseti treatment (Jain et al., 2017). Although these both systems considered as an effective assessment tools, some aspects of prognostic measurements, such as medial and posterior creases, cavus, and heel emptiness values are considered as imperfect (Chu et al., 2010; Bergerault et al., 2013). It may lead the clinicians to perform more casting and unneeded tenotomy procedures (Khan et al., 2017). Therefore, the International Clubfoot Registry Database (ICRD) suggested to record the biomechanical aspects of objective evaluation methods such as measuring the angle of foot positions of four components of clubfoot deformities (cavus, abduction, varus, and equinus) as well as Pirani score (Khan et al., 2017). Due to no agreed standard objective assessment methods for evaluating and monitoring the severities of the clubfoot, many studies suggested that the need of objective assessment method to quantify the severity and predict the prognosis of the treatment (Wainwright et al., 2002; Herd et al., 2004; Siapkara and Duncan, 2007; Ramanathan and Abboud, 2010; Jain et al., 2012, 2017; Fan et al., 2017), and these studies also stated the assessment should be accurate, reliable, and valid to predict the prognosis of the clubfoot. Therefore, we propose this study by using a low-cost 3D scanner to explore the structural changes in clubfoot and quantify the severity of clubfoot.

Another important factor, skin temperature on the clubfoot, need to be considered in the assessment of clubfoot during the casting intervention. The abnormal temperature can be seen in the following conditions such as muscle contracture area, tenderness, pain, swelling, muscle spasm, injury, and other musculoskeletal disorders (Ring and Ammer, 2012) and acute muscle injuries (Schmitt and Guillot, 1984). Most of the ligaments, joints, muscles, and other soft tissues in the clubfoot are contracted toward medial side of the foot (Anand and Sala, 2008). Moreover, the Ponseti method of treatment for clubfoot involves with different techniques: manipulation, casting, Achilles tenotomy, and bracing techniques (Anand and Sala, 2008). One previous study found that the reduction of skin temperature on the palmar side of the thumb after the cervical manipulation (Sterling et al., 2001). However, there is no evidence about the pattern of thermal distribution in clubfoot deformity following before and after manipulation and casting treatment. Some authors reported that the complications of casting techniques such as erythema, pseudoaneurysm (Burghardt et al., 2008), swelling of the toes and forefoot, hyperabduction of midfoot, casting slippage, rocker-bottom deformity (Ponseti et al., 2006), tenderness, pain, mild infection (Hallaj-Moghaddam et al., 2015). It might be due to the reason of casting pressure, improper application of casting or slippage of casting in the treatment phase or improper fitting of foot abduction orthosis in the maintenance phase of Ponseti method. Recently, Infrared thermography (IRT) has been used as popular additional diagnostic tool to assess the skin temperature or pattern of thermal distribution in response to various therapeutic procedures, and musculoskeletal disorders (Bardhan et al., 2015; Packer et al., 2015; Kwok et al., 2017). The in-depth understanding of thermal distribution in clubfoot before and after casting intervention is necessary to increase the success rate of intervention and to reduce the complications. Therefore, a secondary aim of our study is to explore the thermophysiological changes of the clubfoot before and after Ponseti casting in order to determine whether the pattern of temperature distribution changes in response to improvements in foot position as a result of manipulation and casting intervention.

The aim of the study is described as follows: (1) this is a “proof of concept” study looking at the feasibility of the 3D automatic assessment method in assessing (a) the initial severity of the clubfoot and (b) its response to Ponseti manipulation and casting. A reliable, non-invasive 3D assessment method to evaluate clubfoot severity and its response to treatment could be developed. (2) A secondary aim of this study is to explore the relationship, if any, between the thermophysiological changes in the normal foot and the clubfoot at each stage of casting and develop an image classification system of clubfoot. The thermal changes between stages of casting will be evaluated and monitored for clubfoot pathological conditions. The abnormality of the thermal distribution from clubfoot patient could provide insightful data for researchers or clinicians.

In accordance with this protocol, several research questions of 3D scanning and infrared imaging will be addressed (i) the accuracy and reproducibility of 3D scanning method for quantifying the severity of clubfoot, (ii) the reliability and reproducibility of 3D scanning method for detection and quantification of the structural changes in clubfoot in response to manipulation and casting intervention, (iii) the possibility of infrared imaging method to identify the predictable patterns of thermophysiological changes in the clubfoot, and (iv) the changes between the normal foot and the clubfoot in relation to thermophysiological functions.

## Methods

### Exploratory study design

This study uses a Kinect 3D scanner and infrared camera to explore the changes in the clubfoot structure and thermo-physiological functional status at each casting stage in clubfoot intervention. The study registration number is HREC/16/SCHN/163. The study has been reviewed and approved on 17 August 2016 by the Sydney Children's Hospitals Network Human Research Ethics Committee, Sydney, Australia.

### Participants/study population

A total of 10 clubfoot children who are <2 years old will be recruited from The Children's Hospital at Westmead, Sydney. Data will also be collected from the unaffected feet of a total of 10 children with unilateral clubfoot for a normal foot reference. The *inclusion criteria include:* (a) idiopathic congenital clubfoot, (b) both genders, (c) bilateral and unilateral clubfoot, and (d) under 2 years old with untreated clubfoot. The *exclusion criteria include:* (a) a treated congenital clubfoot, (b) clubfoot associated with other neurological conditions, and (c) children over the age of 2 with untreated clubfoot.

### Procedures (recruitment, consent, and data collection)

**Figure 1:**
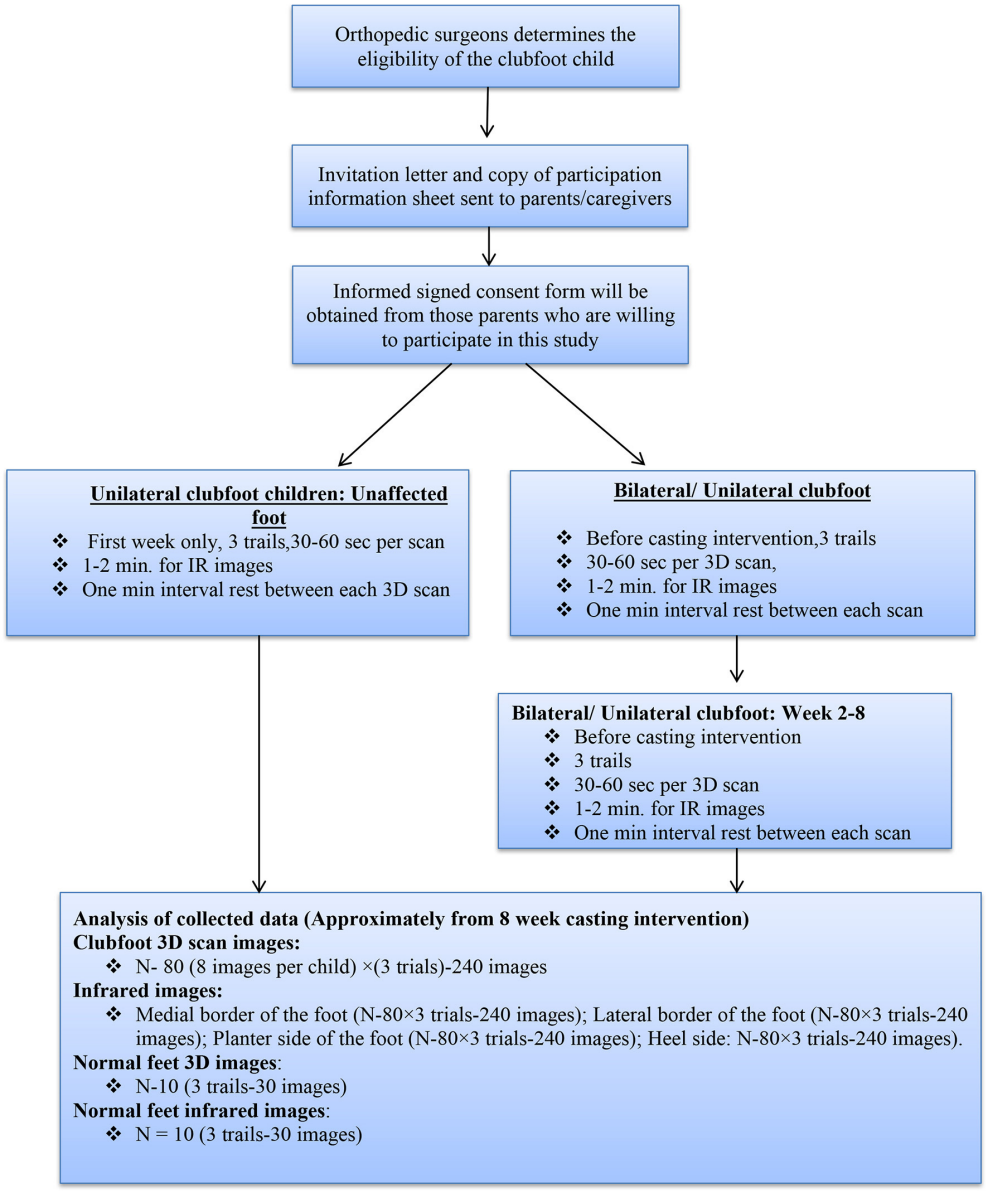
Flowchart of study method.

A total of 20 subjects with untreated clubfoot (*N* = 20; 10 clubfoot; 10 normal feet) will be recruited for this study. Data will also be collected from a total of 10 unaffected feet, from those children with unilateral clubfoot to obtain the reference of a normal foot. In order to allow the parents to make an informed decision on the participation of their child, we will send them an invitation letter and a copy of the participation information sheet prior to their clinical visit. The aims and objectives of the research study, risk and any harmful effects of the equipment, and procedures will be explained to them. If the parents decide to allow their child to participate in the study, a consent form will be provided which is to be signed at their first clinical visit. After the signing of the consent form has taken place, demographic details will be collected from the participants, such as age, gender, and whether the clubfoot is unilateral or bilateral. The following flow chart illustrates the method of this study (Figure 1).

### Equipment/measurement tools

In this study, we use a Kinect 3D scanner and infrared camera as the assessment tools; both pieces of equipment are similar to a video camera. The pictures/3D images will be taken at a distance of ~30–50 cm from the child's foot. The scanner does not make contact with the child during the scanning process. In addition, the scanner/camera does not emit radiation. There are no risks to the patients, parents/caregiver, or clinicians.

### Equipment/outcome measures

#### Kinect 3D scanner

**Figure 2:**
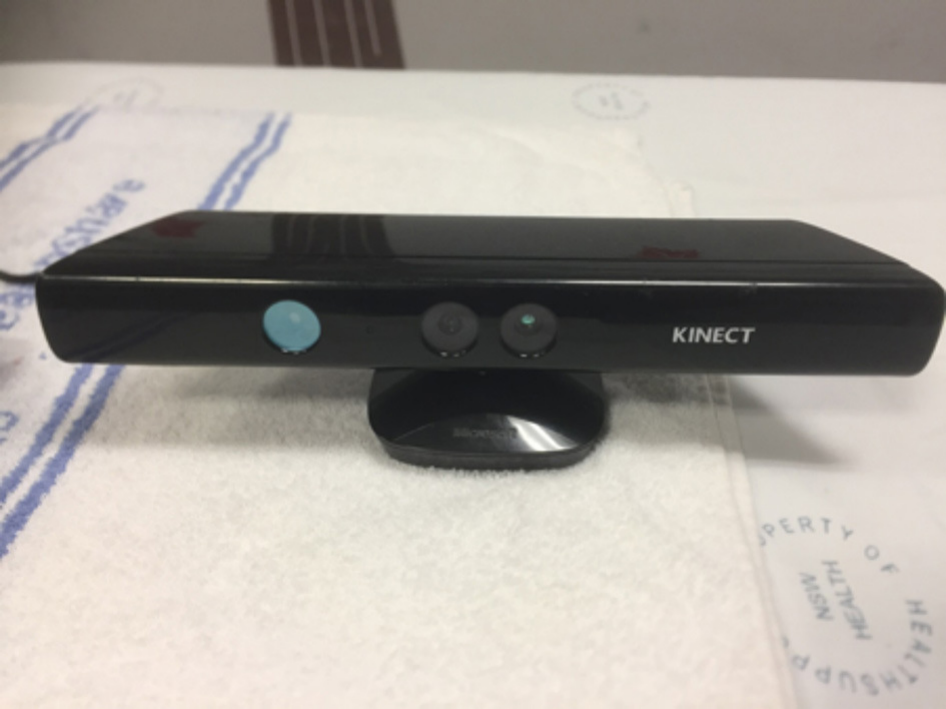
Kinect.

In 2012, Microsoft Company released the Kinect Xbox for playing video games (Figure 2; Taha et al., 2014). Nowadays, it is used for other purposes, for instance, as a 3D measurement tool to obtain accurate anthropometric measurements from normal feet, and clinical analysis tool in the medical and rehabilitation areas (Taha et al., 2014). Also, it is an inexpensive 3D scanning tool which has been established to have high accuracy (1 mm). Therefore, the Kinect 3D scanner is used to collect 3D images from the clubfoot in this study and quantify the severity of the clubfoot.

A number of previous studies have reported that the Kinect is a reliable and valid measurement tool in the clinical setting. For example, Huber et al. (2015) reported that the Kinect is a highly reliable (ICC0.76–0.98) and valid measurement tool for measuring the angles of the shoulder joints. The concurrent validity of the study showed that the accuracy of the Kinect is ±5° in comparison to other measurement tools with a 95% limit of agreement. Another study by Mentiplay et al. (2013) examined the reliability and validity of the Kinect for measuring static foot posture and showed moderate to good intra-rater reliability (ρ = 0.62–0.78) with the Foot Posture Index test and moderate to good correlations (ρ = 0.51–0.85) with the 3D motion analysis (3DMA) system. Mentiplay et al. (2013) also concluded that the Kinect is a more reliable measurement tool to obtain the majority of foot posture measurements as opposed to traditional methods. Also, they reported that the Kinect is more valid than the 3DMA to measure foot posture. Moreover, Kinect 3D imaging can be used as an automated and accurate anthropometric measurement tool to evaluate the body composition, shape, surface area, and volume (Soileau et al., 2016).

#### Infrared camera (FLIR systems)

**Figure 3:**
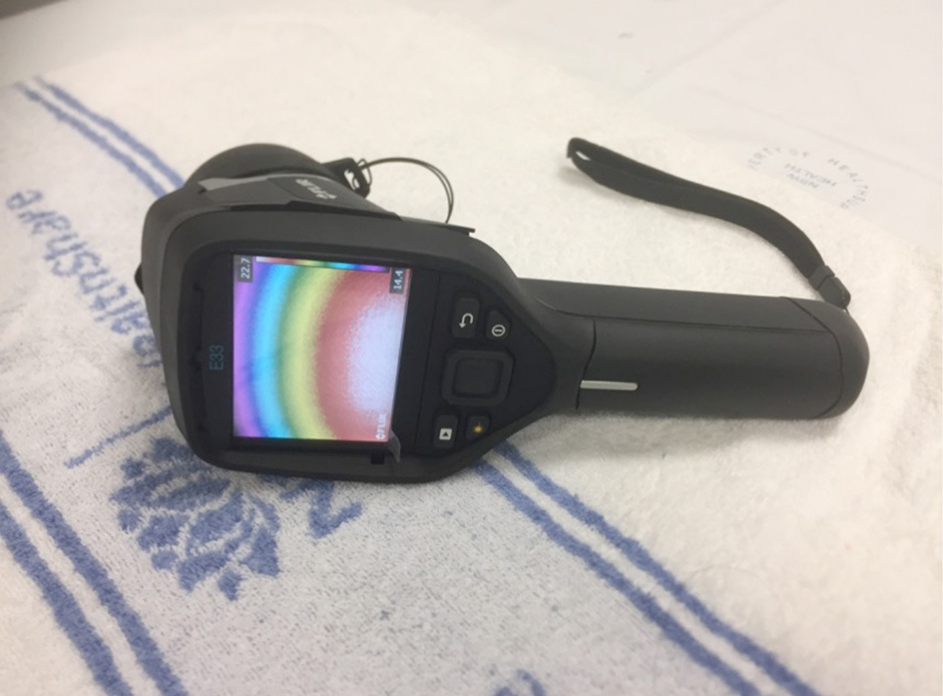
FLIR infrared camera.

Infrared cameras have been widely used as a diagnostic tool in the medical field, especially for dermatological problems, orthopedic and neurological disorders, vascular problems, urological disorders, fever, and breast cancer (Ishigaki et al., 1989; Ng and Acharya, 2009; Hildebrandt et al., 2010). IRT can be used to evaluate the abnormalities in skin temperature on the affected areas of the body, and is also useful on areas of inflammation by calculating the thermal distribution on the skin. To the best of our knowledge, very few studies has been conducted to quantify the distribution of skin temperature of normal foot (Uematsu et al., 1988; Niu et al., 2001; Sun et al., 2005; Zaproudina et al., 2008; Vardasca et al., 2012). Oliveira et al. (2016) proposed a grading and diagnostic system for ankle sprains by using thermography. However, no studies have been conducted on the temperature distribution of clubfoot. FLIR model infrared camera (E33 FLIR model) was adapted for this study. The accuracy of the thermal sensitivity/noise equivalent temperature difference (NETD) of the FLIR model infrared camera (E33 FLIR model) is <0.07°C @ +30°C (+86°F)/70 mK and the accuracy is ±2°C (±3.6°F) or ±2% of reading. It is also reported that IRT is a reliable and valid tool to measure the thermophysiological changes of various conditions. The validity of the IRT has been well demonstrated in diagnosing reflex sympathetic dystrophy, injuries and a number of pathological problems (Bruehl et al., 1996), dermatological issues (George et al., 2008), and diabetes mellitus (Sivanandam et al., 2012). For example, Burnham et al. (2006) reported that an infrared thermometer demonstrates good validity (ICC = 0.92). Moreover, IRT has demonstrated good reliability in several studies, such as a study on reflex sympathetic dystrophy (ICC = 0.94) by Oerlemans et al. (1999); wrist arthritis (ICC = 0.94) by Spalding et al. (2008), and spine, (ICC = 0.95–0.97) by McCoy et al. (2011). In this study, a FLIR infrared camera (Figure 3) will be used to analyze the changes in the physiological functions of the clubfoot at each stage of the casting intervention.

### Data collection procedures

After obtaining informed consent form the participants, the following steps will be followed:

#### Assessment 1

The child will be positioned in a lying/long-leg position in a baby bed. A small baby bed will be positioned in the standard hospital bed. The small baby bed has a “V” shape and is perpendicular at the end. The “V” shape of the bed is to maintain the child in a supine position with ~20–30° hip flexion and 30–45° knee flexion. It will provide comport and support to the baby in this position. After that, a small color sticker will be used to identify 6 landmarks (medial malleolus, lateral malleolus, first and fifth metatarsal heads, talus head, and heel point). The parent of the child will be requested to hold the knee of the child for 30 s. Then, the 3D camera will be used to move around the foot (360°) to capture the 3D images. To capture the 3D images of the right side of the foot, the 3D scanner will be moved from the medial border of the clubfoot in a clockwise direction. To capture the 3D images of the left side of the foot, the 3D scanner will be moved from the medial border of the clubfoot in a counter-clockwise direction. The capturing of the 3D images requires 30–60 s. The marking of the anatomical landmark with a small color sticker requires 1–2 min. Three trials of 3D scanning will be carried out to establish the reliability and validity of the instruments. The child is given a break of 1 min to rest between each trial. The 3D scanning will be performed weekly (once a week) before each casting. In total, 6–8 scannings (up to the last casting including before and after the Achilles tenotomy) will be collected for each participant.

The same procedures will be done for the normal/unaffected foot. However, the scanning will be only done once for the normal foot during the first week of the hospital visit.

#### Assessment 2

The child will be positioned in a lying/long-leg position in a baby bed. The investigator will manually hold the infrared camera to take infrared images of the following areas of the clubfoot: medial, lateral, heel, and plantar sides of the foot. In this study, the following protocol guidelines will be followed to obtain the thermal images. The temperature of experiment room will be maintained 22–26°C. To avoid the high temperature due to the casting on the affected feet, the experiment will be performed after 15 min of casting removal. The duration of this experiment procedure requires 1–2 min. Infrared images will be collected weekly (once a week) before each casting. Three trials will be collected to establish the reliability and validity of the instruments. The same procedures will be done for the unaffected foot. However, the infrared imaging will be done only once for the normal foot and 3 trials.

## Data analysis and statistics

### Method of 3D image analysis

The collected 3D scanned images will be stored in the computer as obj or stl files and then processed by using Artec software to create 3D images of the foot. The following methods of data analysis will be done to find out the structural changes of the clubfoot at each stage of casting, and predict the progress of the casting intervention.

**Figure 4:**
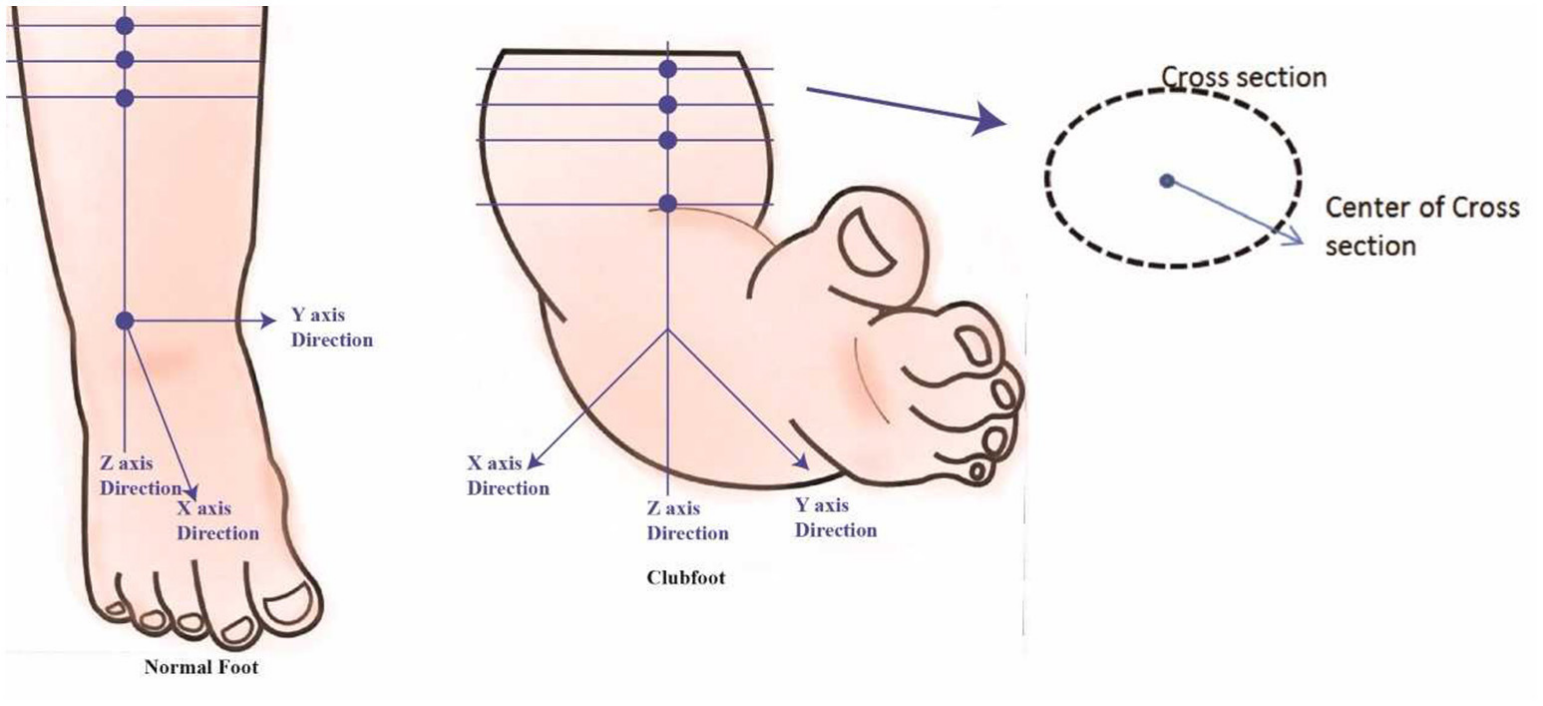
Foot alignment.

The data analysis includes 3D foot alignment in the x, y, z coordinates system, cross-sections, parameter estimations, and curvature analysis will be done in the clubfoot (Figure 4). The cross section, parameters and curvatures will be compared to the 3D data of children with a normal foot. The z-axis will be based on the center of the lower foot region. After a manual alignment, the cross sections of the lower leg at 5 mm (or the distance can be adjusted) will be done. Using the 3D scanned data, for each cross section, the center of the points will be calculated. Based on the center of the points, a linear regression is plotted to create the lower center as the z-axis direction. The X-axis direction is based on the direction of the knee cap. The Y-direction is based on the direction from the center to the medial side. The center point or zero point is estimated at the ankle joint position.

### Methods of cross-sections, parameters estimation, and curvature analysis

**Figure 5:**
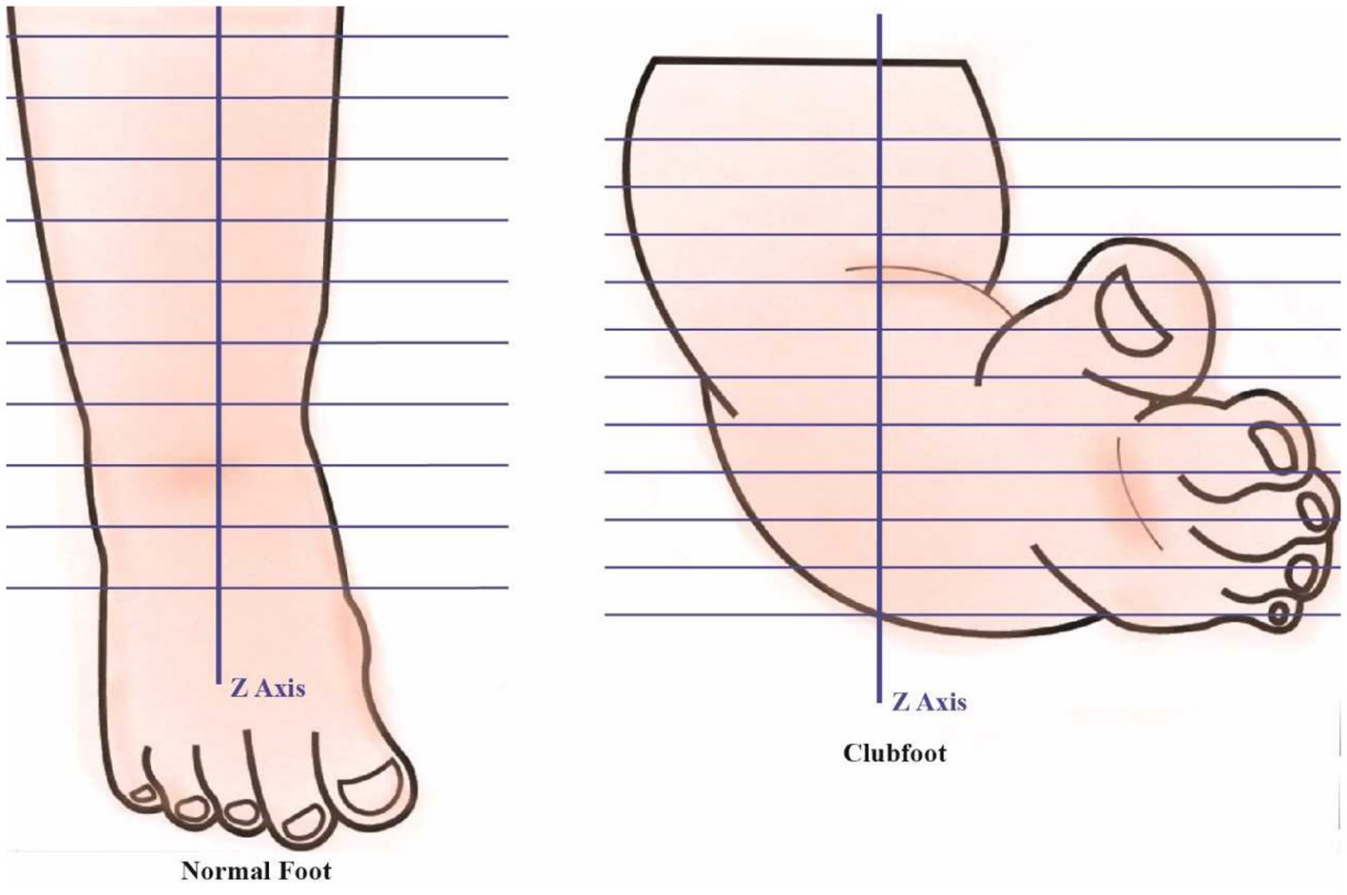
Cross sections.

Along the Z-Axis, cross sections are created at every 5 mm (or the distance can be adjusted). The different cross sections will be used to see differences between each casting stage of the clubfoot and deviation from normal feet. Each cross-section parameter, such as length, width, and the radial distance from the center to the cross-section edge for specific angles (15°, 30°, 45°, 60°….) is also calculated (Figure 5). Similarly, cross sections are computed along the X-Axis and Y-Axis. These parameters will be compared to determine the different levels of deviation for different types of clubfoot.

The parameters and curvatures based on sampling will be used to compare between the different types of clubfoot with a normal foot. In addition, some anatomical landmarks are marked while scanning. The position of the anatomical landmarks with respect to the coordinate system will also highlight the differences between the various types of clubfoot with respect to normal feet.

#### Statistics for 3D images

Descriptive statistical analysis will be conducted to calculate the mean and standard deviation for the child's age, gender, and type of clubfoot. One way repeated measure ANOVA will be used to analyze the effectiveness, and monitor the progress of the casting intervention. The following parameters will be interpreted from the 3D scanning images to achieve the aims of this explorative study (Table 1): Angles, cross section parameters such as length, width, and the radial distance, and distance between the anatomical landmarks (medial border and lateral border of the foot, and distance between the talar head and medial malleolus).

**Table 1:** Parameters from 3D imaging analysis and infrared imaging.

**S. no**	**Parameters from 3D and thermography assessment method**
**PARAMETERS FROM 3D IMAGES**	
1	Angles
2	Length
3	Width
4	Radial distance
**DISTANCE BETWEEN THE ANATOMICAL LANDMARKS**	
5	Medial border of the foot
6	Lateral border of the foot
7	Distance between the talar head and medial malleolus
**PARAMETERS FROM INFRARED IMAGE**	
8	Foot skin temperature

Cross-sections, parameters estimation, and curvature analysis will be conducted to calculate the length, width, and the radial distance (mm) from the center to the cross section edge for specific angles of the clubfoot (15°, 30°, 45°, 60°,….) and followed by linear regression will be conducted and plotted as described in the “Method for 3D image analysis.” Then SPSS will be used to compute the descriptive statistics to do the following analyses, such as mean and standard deviation. The mean and standard deviations (parameters: length, width, radial distance) will be calculated to find out the structural changes and progress of the clubfoot intervention at each casting. In addition, descriptive statistics (mean and standard deviation) will be computed by SPSS for the distance of the anatomical landmarks [in 3D image data, the distance of the anatomical landmarks will be measured between the medial malleolus (MM) to the first metatarsophalangeal (MTP) joint, lateral malleolus (LM) to the 5th MTP joint and distance between the lateral head of the talus, (MM and LM) to predict the changes of the foot].

**Figure 6:**
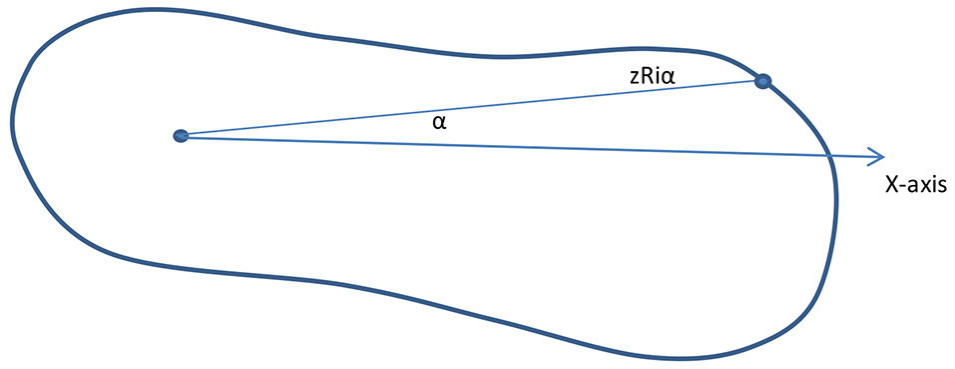
Parameter estimation and curvature (example, section i).

The initial average range of the parameters of length, width, and radial distance and final average score of intervention will be used to calculate the required number of castings, and these parameters will be compared to show the different levels of deviation for the different types of clubfoot. In addition to the parameters, the foot shape can be sampled and curvatures determined. For example, as shown in Figure 6, the radial length is zRiα when the angle is α for section I and the cross section along the z-axis. The curvature can be calculated by using different values for different angles. In addition, an automatic classification system will be developed from the 3D images of the clubfoot by using feature extraction and support vector machine (SVM) classifier model by using MATLAB.

#### Infrared image analysis and statistics

##### Parameters

The skin temperature of the foot on the selected region of interest (ROI), will be interpreted at each stage of the clubfoot intervention. The skin temperature of the clubfoot will be compared with selected areas of the foot (forefoot, mid foot, and hindfoot). The temperature of the ROI of the clubfoot will be compared with the skin temperature of the normal foot. Descriptive statistics and an independent *T-*test will be conducted in SPSS to calculate the differences in skin temperature between the normal foot and clubfoot. In addition, automatic classification of clubfoot will also be developed by using MATLAB image processing methods and machine learning techniques.

### Statistics for infrared image analysis

The following steps will be used to analyze the infrared images in MATLAB. The Kruskal–Wallis test or Shapiro–Wilk test will be used to analyze the normal distribution of the collected data. The first step of image analysis is data acquisition. In the second step, an algorithm for 3 areas (forefoot, mid-foot, hindfoot) will be developed to automatically detect the ROI. A Canny edge detection operator and gradient operator will be used to segment the ROI into the forefoot, mid-foot, and hind-foot. In the third step, the image segmentation process will be done for 3 selected (ROI) areas of the clubfoot. Then, the final step is the feature extraction of thermal images of the foot. Statistically, feature extraction will be performed by using higher order statistical features, cross section and histogram generation will be done for the selected ROI. Descriptive statistics, skewness, kurtosis, and entropy statistical tests will be used to determine the characteristics of the thermal distribution of selected ROI. Mean and standard deviation will be calculated in descriptive statistics to evaluate the normal distribution of temperature. Entropy statistical features analysis will be done to find out the abnormalities of the segmented thermal images to classify and to find out the severity of the clubfoot. For the automatic classification of clubfoot, the SVM classifier model will be used to classify the clubfoot into different categories such as mild, moderate, or severe. For accuracy, the sensitivity and specificity will be tested by receiver operating characteristic (ROC) analysis such as sensitivity and specificity.

## Discussion

Clubfoot management is still challenging in terms of its assessment and intervention methods. The accurate evaluation of clubfoot is essential for assessing the initial severity, choosing the treatment options, monitoring and predicting the progress of the intervention, and predicting relapses of the foot (Wainwright et al., 2002; Herd et al., 2004; Siapkara and Duncan, 2007; Ramanathan and Abboud, 2010; Jain et al., 2012, 2017; Fan et al., 2017). There are several scoring systems that have been used as assessment tools to evaluate the severity of clubfoot deformity. But there is no universal standard assessment method available to quantify the initial severity of the clubfoot (Jain et al., 2001; Gigante et al., 2004; Dyer and Davis, 2006; Ramanathan et al., 2009; Ramanathan and Abboud, 2010; Yapp et al., 2012).

To the best of our knowledge, our study is the first study tries to evaluate the initial severity of the clubfoot by using a Kinect 3D scanner and IRT imaging methods. The primary aim of this study is to develop an automatic evaluation system to assess the structural changes of the clubfoot and quantify the severity of clubfoot. The following parameters will be collected to assess the initial severity of the clubfoot: Angles, cross section parameters such as length, width, radial distance, distance between the anatomical landmarks, and temperature of the foot skin.

The proposed novel 3D assessment method can be used as an objective measurement method for quantifying the severity of clubfoot at each stage of casting intervention (treatment phase) and maintenance phase of Ponseti treatment methods. The main advantages of using Kinect scanning is low cost, high efficiency, non-radiating equipment, and its reliability and it can be can be used in remote or developing areas as well as hospital settings. This method can be applied without sedation in newborn babies with clubfoot. However, this study has some limitations. Acquisition of 3D images from the feet by using Kinect scanner is quite difficult procedure than other parts of the human body especially in feet of new born babies. The plantar side of foot cannot be visible in a standing position of children or adults while performing the Kinect 3D scanning. Similarly, the 3D image of heel and ankle sides of foot is not visible in the lying or long leg position of the new born babies. To overcome this barrier, a “v shape” small (baby bathing) bed is selected for positioning the baby and obtaining the full shape of the foot as described in section Data Collection Procedures. In addition, two trained persons are required to acquire the 3D images of clubfoot successfully because one person is needed to handle the Kinect camera while other person need to take care of controlling the computer and Artec studio software. A constant moving speed and distance (~50 cm between the clubfoot and Kinect camera) around foot should be maintained during the scanning procedures to obtain the 3D images of the foot. Too far or close to the objects, the Kinect scanning software will interfere with scanning or create noisy 3D images. Only one hospital site is used in this study for data collection. Therefore, the study design can be time consuming. Also, further ethical approval is needed to extend the work to another hospital site.

The significance of this study is to develop a low-cost 3D automatic assessment method to quantify the initial severity of the clubfoot and monitor and predict the structural changes of the clubfoot at each casting of clubfoot intervention which can be helped to predict the number of castings required to normalize the foot. It will also be useful to assess the severity of clubfoot in remote areas and areas where medical expertise is unavailable. In addition, the outcomes of this recording and monitoring approach will be useful to do further research to design comfortable braces to avoid the relapse of the clubfoot.

## Ethics and dissemination

The study has been reviewed and approved by the Sydney Children's Hospitals Network Human Research Ethics Committee, Sydney, Australia and the HREC reference number: HREC/16/SCHN/163. The HREC is accredited by the NSW Department of Health (New South Wales, Australia) as a lead HREC under the model for single ethical and scientific review, and by the National Health and Medical Research Council (NHMRC) as a certified committee in the review of multi-center clinical research projects. The ethical approval for this study is valid for up to 5 years and was assessed and approved in accordance with, and meets the requirements of, the National Statement on Ethical Conduct in Human Research (2007). Any changes or modifications in the protocols will be submitted to the HREC for approval. After ethical approval, SSA approval was obtained and the SSA reference number: SSA/16/SCHN/390. In that SSA, Kinect and Infrared camera were declared as non-ionizing radiation equipment. In addition, the principal investigator GB was instructed to take X-RAY and Mantoux test to avoid infection to children and other workers in the hospital. An information sheet has been given to the parents of the children and also adequate time has been provided to them to take the decision to participate in this study. The findings of the study will be disseminated for the first author's Ph.D. thesis. Furthermore, we are planning to publish the outcomes of this study in other peer-reviewed journals and conferences for widespread dissemination.

## Trial status

This trial is currently recruiting clubfoot subjects from Children's Hospital at Westmead in Sydney, Australia.

## Author contributions

BG is the main researcher of this study and studying for a fulltime dual Ph.D. degree at The Hong Kong Polytechnic University and the University of Technology Sydney Australia. and responsible for the study concept and drafted the full manuscript and protocols. AL, AA-J, and JY are the chief supervisors of BG. AL, AA-J, and JY reviewed and edited the manuscript. AL is responsible for funding the 3D and imaging equipment. JY is responsible for funding of publications. PG and AC contributed clinical expertise while designing the study protocols for an ethical process. BG will collect the data from Children's Hospital at Westmead in Sydney, and PG and AC will help to recruit clubfoot children at the Children's Hospital at Westmead in Sydney, Australia. BG is responsible for performing the experiment and collecting the data from the hospital. BG and AL will conduct statistical analysis for 3D and infrared imaging. BG, AA-J, and AL will conduct analysis for developing automatic assessment method with the use of imaging. All authors (BG, AL, AA-J, JY, PG, and AC) reviewed, edited, and approved the final manuscript.

### Conflict of interest statement

The authors declare that the research was conducted in the absence of any commercial or financial relationships that could be construed as a potential conflict of interest.
